# Hand Involvement and Its Association with Burn Characteristics, Surgical Management, and Length of Stay in Paediatric Inpatients: A 10-Year Cross-Sectional Study from Western Australia

**DOI:** 10.3390/ebj7020023

**Published:** 2026-04-30

**Authors:** Lachlan James Madge, Lisa J. Martin, Emma Catherine Mill, Fiona M. Wood, Tiffany L. Grisbrook

**Affiliations:** 1 University of Western Australia Medical School, Faculty of Health and Medical Sciences, The University of Western Australia, Perth, WA 6009, Australia; lachlanjames.madge@health.wa.gov.au (L.J.M.); tiffany.grisbrook@health.wa.gov.au (T.L.G.); 2 Burn Injury Research Unit, School of Biomedical Sciences, The University of Western Australia, 35 Stirling Highway, Crawley, Perth, WA 6009, Australia; fiona.wood@health.wa.gov.au; 3 Fiona Wood Foundation, 11 Robin Warren Drive, Murdoch, Perth, WA 6150, Australia; 4 Curtin School of Allied Health, Faculty of Health Sciences, Curtin University, Kent Street, Bentley, Perth, WA 6102, Australia; emma.mill@health.wa.gov.au; 5 Telethon Kids Institute, Nedlands, Perth, WA 6009, Australia; 6 Perth Childrens Hospital, Nedlands, Perth, WA 6009, Australia

**Keywords:** paediatric burns, hand burns, burn epidemiology, length of stay

## Abstract

**Highlights:**

**What are the main findings?**

- Paediatric isolated hand burns occur in younger children and are more often caused by contact or friction injuries.
- Hand involvement did not independently prolong length of stay; however TBSA, flame injury, surgery, and rural residence independently did.

**What are the implications of the main findings?**

- Length of stay is driven by injury severity and management rather than hand burn location.
- The impact of hand burns may relate more to functional and rehabilitation needs than acute admission duration.

**Abstract:**

Background: Hand burns are a key criterion for immediate referral to tertiary burn centres in Australia, New Zealand, and internationally, yet few studies have examined how paediatric burn epidemiology, surgical management, and length of stay (LOS) differ according to the extent of hand involvement. The objective of this study was to describe and compare the demographic profiles, burn injury characteristics, and clinical management between three groups: children with (1) burns involving only the hands, (2) burns involving the hands and other sites, and (3) burns not involving the hands who were admitted to the paediatric Burns Service of Western Australia (BSWA) over a 10-year period. Methods: This cross-sectional study included all burn admissions to the state paediatric burn unit between July 2012 and June 2022. Descriptive statistics and univariate regression used to compare groups. A multivariate log-linear regression model was used to assess the independent association between hand involvement and length of hospital stay, adjusting for identified confounders. T Results: Children with burns isolated to the hands were younger, had a smaller percentage of total body surface area (%TBSA), were more likely to have sustained contact or friction burns, and were more likely to undergo skin grafting procedures compared to those with burns involving the hands and other sites, and those with burns not involving the hands. Despite these differences, hand involvement was not identified as an independent predictor of initial LOS. Conclusion: Paediatric patients with hand burns did not have longer initial hospital admissions than those without hand involvement. Future research needs to assess longer term impacts of hand burns.

## 1. Introduction

Paediatric burn injuries represent a frequent reason for presentation and admission to tertiary burns centres [1,2]. In 2021, the global incidence of burn injuries in children under the age of 15 was estimated at around 2.76 million [3]. They represent the fifth most common non-fatal injury and the third-leading cause of trauma-related mortality in children and are associated with the greatest length of stay (LOS) among all paediatric hospital trauma admissions [4,5].

In Australia and New Zealand, there were 735 paediatric patients admitted to 17 burns units for more than 24 h or for burn-related surgery between 1 July 2022, and 30 June 2023 [6]. The median age of injury for these children was three years, with a male predominance of 60.5% [6]. Scalds were the most common mechanism of injury, and the median total body surface area (TBSA) affected was 2.0% [6]. These trends in paediatric burn epidemiology largely reflect those published in international literature [7,8,9,10,11,12,13,14,15,16].

Hand burns are particularly common in children, largely due to their natural inclination to explore through tactile engagement, along with their limited understanding of thermal hazards [4,17,18,19]. Their slower withdrawal reflexes and thinner palmar skin compared to adults make them more susceptible to deeper, more severe burn injuries [4,19,20]. Such injuries can lead to significant impairments in both function and appearance, with lasting effects on quality of life [19,21,22,23,24,25,26,27,28].

Burns involving special areas, such as the hands, are a recognised criterion for immediate referral to tertiary burn centres in Australia, the United States, and the United Kingdom [29,30,31]. However, contemporary evidence on the specific impact of hand burns on clinical outcomes in paediatric patients remains limited. Dodd et al. reported that hand involvement in children with extensive burns was associated with longer hospital stays, prolonged intensive care unit (ICU) admissions, extended mechanical ventilation, and a greater number of surgical procedures [32]. However, no distinction was made between outcomes for children with isolated hand burns and those with burns involving multiple sites, including the hands [32]. This distinction is particularly important given the high prevalence of isolated hand burns in children.

To address this gap in the literature, this study had two key objectives. The first objective was to describe the epidemiology—including demographic profiles, burn injury characteristics, and hospital LOS—and the burn management procedures of all paediatric patients admitted to the Burns Service of Western Australia (BSWA) over a 10-year period. The second was to compare these variables across three groups: (1) children with burns isolated to the hands; (2) children with burns involving the hands and other sites; and (3) children with burns not involving the hands. This analysis aimed to clarify the specific impact of hand involvement on the epidemiology of paediatric burn patients.

## 2. Materials and Methods

This study was a cross-sectional analysis of all paediatric patients (0 to <16 years) admitted to the paediatric BSWA with burn injuries between 1 July 2012, and 30 June 2022. All data were extracted from a local collection of paediatric BSWA burn data at Perth Children’s Hospital (PCH) in May 2024. This included data for all burns patients admitted to the Princess Margaret Hospital (PMH) up until 2018, when it was replaced by PCH. For the comparative analysis, patients were divided into three groups: Group 1 included children with burns isolated to the hands, Group 2 included those with burns involving the hands and other sites, and Group 3 included children with burns not involving the hands.

Ethics approval for this study was obtained from the Child and Adolescent Health Service (CAHS) Human Research Ethics Committee, based at Perth Children’s Hospital, Western Australia (RGS5672). Data is not made publicly available due to ethical restrictions. The research was conducted in accordance with the ethical principles outlined in the World Medical Association’s Declaration of Helsinki and followed the STROBE (Strengthening the Reporting of Observational Studies in Epidemiology) guidelines for reporting cross-sectional studies [33,34].

### 2.1. Study Population

The local collection of paediatric BSWA data included data for all patients transferred or admitted to PMH or PCH within 28 days of injury, where the primary reason for admission was a burn, and at least one of the following conditions was met: either the patient stayed in hospital for 24 h or longer; or the patient underwent a burn wound management procedure in the operating room. Data were also captured for any subsequent readmissions to a specialised burn unit within 28 days of discharge from the initial admission.

Data were not available for patients admitted for treatment of non-burn skin disorders (e.g., Stevens–Johnson syndrome, toxic epidermal necrolysis, graft-versus-host disease), patients who had died prior to arrival at the hospital, and those with erythema only not deep enough to qualify as superficial dermal burns.

### 2.2. Data Collection

Demographic variables included patient age at the time of injury, sex, and area of residence, which was categorised as metropolitan, rural, overseas, or other/unknown according to Australia Post’s Domestic Delivery Estimator [35]. Burn characteristics recorded included the mechanism of injury (e.g., scald, contact, flame, friction, other), affected TBSA (%), and the anatomical location of the injury (hands only, hands plus other site(s), or other site(s) only). Burn depth was recorded as superficial dermal, mid-dermal, deep dermal, or full thickness. Clinical data included LOS in days, as well as details of burns management procedures performed in the operating theatre. For patients who underwent such procedures, the type of intervention was recorded and categorised as having received either skin cell products only (i.e., ReCell^®^ (Avita Medical, Valencia, CA, USA)), skin grafting only, a combination of both, or another type of procedure. Admissions to the intensive care unit were also recorded, where present.

### 2.3. Statistical Analysis

All statistical analyses were conducted using Stata statistical software v16.1 [36]. A significance threshold of *p* < 0.05 was applied throughout. Descriptive statistics were computed for the overall patient cohort and stratified by study group. Categorical variables are presented using frequencies and percentages, and continuous variables are presented using means with standard deviations or medians with interquartile ranges, depending on data distribution. Between-group differences were assessed using univariate regression analyses. Covariates demonstrating statistical significance in these were subsequently considered for inclusion in a multivariate model.

To refine the multivariate model, a backward elimination approach was employed. As part of the data preparation, percentage of total body surface area (%TBSA) data was log-transformed to address skewness. Intensive Care Unit admission was not included as a covariate in the main regression model due to its rarity (<1% overall) and perfect non-overlap with the ‘hand only’ group, which resulted in quasi-separation. As TBSA was already included in the model, ICU admission was considered a downstream factor and therefore excluded to maintain model stability.

A multivariate log-linear regression model was constructed to assess the independent association between hand involvement and length of hospital stay, adjusting for confounders identified in the univariate analysis. This analysis compared group 1 to group 2. and group 1 to group 3. Backward elimination was used to derive the final model, starting with variables selected based on their association with length of stay in univariate analyses (*p* < 0.2), to reduce overfitting and retain only those covariates contributing meaningfully to model fit. This approach balances parsimony with explanatory power and standard statistical practice for observational studies to refine multivariable models [37]. Missing data were handled in Stata using listwise deletion; therefore, any observation with a missing value in one or more model variables was excluded from the final analysis. Given the use of a negative binomial regression model with robust standard errors, model fit was evaluated using McFadden’s pseudo-R^2^, an appropriate measure of relative explanatory power for non-linear models.

## 3. Results

Descriptive statistics for demographics, burn injury characteristics and clinical data, including overall group comparisons are shown in Table 1.

**Figure 1:**
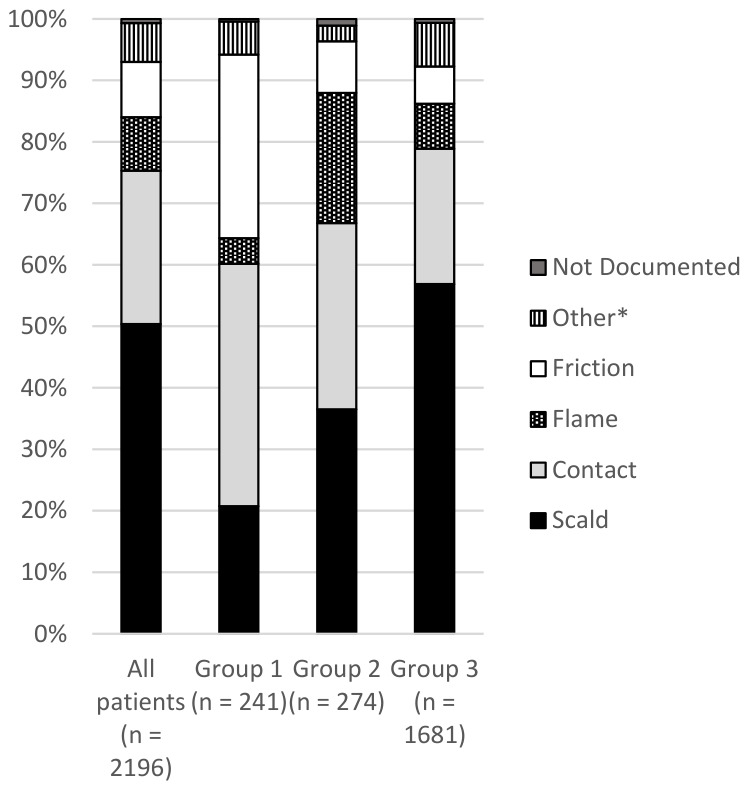
Distribution of the Mechanism of Burn Injury Among All Children and by Hand Involvement Group. Note. Group 1 = children with burns isolated to the hands; Group 2 = children with burns involving the hands and other sites; Group 3 = children with burns not involving the hands. * Other mechanisms of burn injury include chemical, electrical, radiant heat, cooling, pressurised gas/air, and other specified causes.

**Figure 2:**
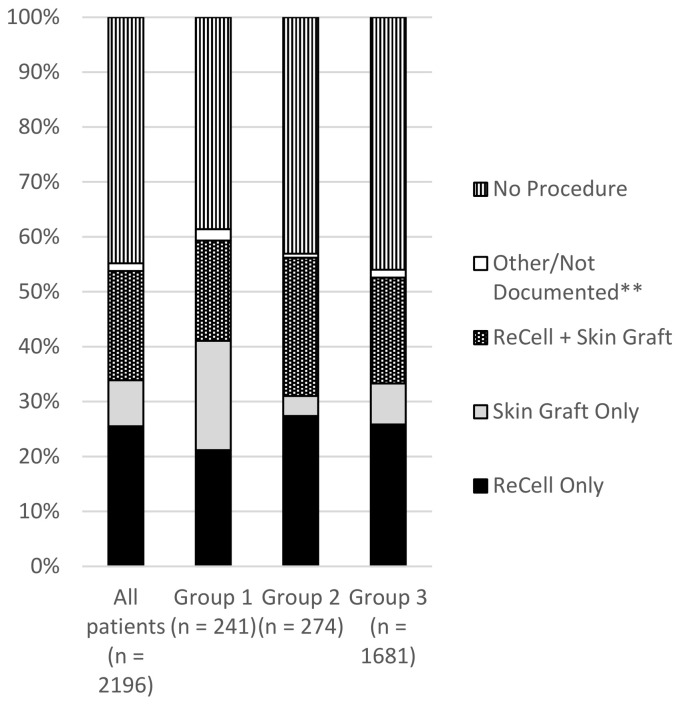
Distribution of Burn Management Procedures in Theatre Among All Children and by Hand Involvement Group. Note. Group 1 = children with burns isolated to the hands; Group 2 = children with burns involving the hands and other sites; Group 3 = children with burns not involving the hands. ** Other burn management procedures in theatre include escharotomy, debridement only, use of temporary skin closure product, use of dermal reconstructive product, allograft, dressing change, fasciotomy, amputation, and other specified causes.

Most injuries (60.93%) occurred in children under five years of age, with 30.16% affecting children under two years of age. Although there was no significant difference in gender or area of residence distribution among the three patient groups, children with isolated hand burns were found to be significantly younger than the children from the other groups, with a median age of 2.3 years (Table 1). The type of burn differed significantly between groups, with contact and friction burns more likely in the hand only group and scalds the most common in the other two groups (Table 1, Figure 1). The other body sites involved were diverse, and included burns to the head and neck, chest and trunk, upper and lower limbs, and feet. Most children (55.19%) underwent a burn management procedure in the operating theatre (Table 1). However, those with isolated hand burns were more likely to receive a skin graft alone compared to other groups (Table 1, Figure 2). In contrast, children with burns affecting both the hand and other sites were more likely to receive a combination of grafting and skin product application (Table 1, Figure 2). No patients with an isolated hand burn were admitted to ICU (Table 1).

### Regression Analyses

Univariate analyses assessed the relationship of the variables shown in Table 1 with LOS. The full output from these regression analyses is detailed in the Supplementary Materials Table S1. The variables that had an unadjusted, significant association effect on LOS were study group, TBSA, surgery type, burn type, and area of residence Importantly, study group had a highly significant association with LOS in the univariate analysis, showing that those burns that only involved the hands had a longer LOS than the other groups when other factors are not accounted for (Table 2).

The variables that demonstrated a relationship with LOS were used in the initial regression model as per standard statistical practice. Following backward elimination, TBSA, burn type, area of residence, and surgery type remained in the final model (Supplementary Materials; Table S2). The study group variable was retained regardless of statistical significance, as it represented the primary variable of interest. The final model, presented in Table 3, showed that study group was not significantly associated with length of stay after adjusting for TBSA, flame burn, area of residence, and surgery type. Model performance was acceptable, with a McFadden’s R^2^ of 0.12 indicating moderate explanatory power, and relatively low AIC (10,442.28) and BIC (10,481.5) values supporting overall model adequacy.

## 4. Discussion

This study described the specific impact of hand involvement on the epidemiology and burn management procedures of children treated for burn injuries at the paediatric BSWA over a 10-year period. While many paediatric burn injuries can be effectively managed in outpatient settings [1,10,19], this research focused exclusively on children admitted to the ward or those who underwent a burns management procedure in theatre.

The demographic and burn-related characteristics observed in this study were largely consistent with patterns reported in the existing literature [7,8,9,10,11,12,13,14,15,16]. As seen globally, male children experienced burn injuries more frequently than females, and the age distribution was notably skewed toward younger children [7,8,9,10,11,12,13,14,15,16]. Those under two years of age had the highest incidence, accounting for over 30% of the study population. In the comparative analysis, although there was no difference in the gender distribution between groups, children with isolated hand burns were found to be significantly younger than children with burns involving the hands and other sites and children with burns not involving the hands, with a median age of 2.3 years. This underscores the vulnerability of younger children to burn injuries, a trend already attributed in the literature to their exploratory behaviours and lack of awareness of thermal hazards [4,17,18,19].

Scalds and contact burns emerged as the two most common mechanisms of injury overall, consistent with international data [8,9,12,13,38,39]. However, few studies have explored how the mechanisms of paediatric burn injuries vary according to hand involvement. Our comparative analysis revealed significant variations in the prevalence of burn mechanisms across groups. Children from Group 1 had the highest prevalence of contact burns (39.42%) compared to children from Groups 2 and 3. This likely reflects specific injury mechanisms, such as touching hot objects like stovetops or oven doors. Supporting this, a cross-sectional review of 1215 children by Kemp et al. found that 83% of contact burns in children under five were caused by touching hot items, with 67% affecting the hands and only 11% involving multiple sites [9]. This finding provides a plausible explanation for the high prevalence of contact burns in children with isolated hand injuries in our cohort.

Friction burns were also disproportionately common among children with burns isolated to the hands (29.88%), compared to children with burns involving the hands and other sites (8.39%) and children with burns not involving the hands (6.07%). This disparity reflects the typical mechanisms of friction injuries. In a study of 421 paediatric patients, Ahmed, Cheng, and Burd reported that nearly 80% of treadmill-related friction burns involved the hands—the most frequently identified cause of friction burns in children, as also noted by Kemp et al. [9,40]. This again highlights the susceptibility of the hands to burn injuries in such scenarios.

Conversely, scald injuries were most prevalent in children with burns not involving the hands (56.87%) and those with burns involving the hands and other sites (36.5%). This likely reflects mechanisms such as pulling hot liquids or food down onto themselves, which typically resulting in burns to the face and upper trunk rather than isolated hand injuries [9]. Kemp et al. found that nearly half (48%) of scald injuries in children occurred this way, further supporting our findings and explaining the lower prevalence of scalds in children with burns isolated to the hands [9].

Mid-dermal burns were the most commonly reported depth across all groups, followed by deep dermal and full-thickness burns. While the distribution of burn depth did not significantly differ between groups, notable differences were observed in the %TBSA estimates. Unsurprisingly, children with burns isolated to the hands had significantly lower %TBSA estimates compared to children from the other groups. Systemic illness and comorbidities were not assessed in our study, however hand involvement has been suggested to be an indicator of more severe illness and a greater requirement for resources for the same TBSA [32].

We also examined the burn management procedures performed in theatre across the study population. In line with Stockton, Harvey, and Kimble, who reported that 52% of paediatric inpatients required surgery, we found that 55.19% of our patients underwent at least one operative procedure [38]. Children with burns isolated to the hands were significantly more likely to undergo skin grafting alone, whereas those with burns involving the hands and other sites more frequently underwent combined grafting and skin substitute application. These differences might reflect that smaller burns might not require autologous skin cell application, and the larger burns might be deemed to necessitate this to optimise wound healing and scar outcome.

Despite the differences found in the epidemiology and burn management procedures between groups, no significant relationship was identified between hand involvement and LOS when controlling for the dependent variables identified in the univariate regression analyses, such as %TBSA and mechanism of injury. This finding suggests that hand involvement, while associated with distinct injury patterns and management approaches, does not independently influence the duration of hospitalisation during the acute phase of care.

Some limitations must be considered when interpreting these findings. First, data were available for patients admitted for more than 24 h or who underwent a burn wound management procedure in theatre. This inherently biases the sample toward more severe injuries requiring hospital-based care, underrepresenting the epidemiological and clinical characteristics of less severe burns managed in outpatient settings. As a result, the findings are not generalisable to the broader outpatient population, which comprises the majority of paediatric hand burn cases [1,10,19,38]. In addition, this analysis does not account for natural changes in LOS over time due to alterations to the standard model of care. Despite surgical intervention types being stable over the period, it was shown that there was an 1.8% annual reduction in LOS for all burn patients in WA between 1983 and 2008 after adjustment for burn severity and other factors [41].

Second, missing data may have affected the reliability and completeness of the analysis. Burn depth was excluded from the regression model due to missing data in approximately 31% of cases. Because Stata applies listwise deletion by default, any observation with missing values in any model variable—whether the outcome or a predictor—is omitted from the analysis. Although burn depth is likely to influence length of stay, its exclusion was necessary to preserve sample size and minimise potential bias introduced by listwise deletion. Additionally, ICU admission data were excluded due to the small sample size (n = 20), which prevented adjustment for ICU status when analysing length of stay. This may have biased the findings towards less severe cases, potentially underestimating the impact of hand involvement on hospitalisation duration. Finally, some potentially important confounding variables—such as the presence of complications like infection—were not systematically documented in the registry and were therefore excluded from regression analyses. This may have introduced bias in interpreting associations, particularly with respect to LOS.

Third, the generalisability of these results to other tertiary burns centres may be limited. Prior research has highlighted considerable variability in paediatric burn management across centres in Australia and New Zealand [42]. Differences in admission criteria, surgical thresholds, and postoperative care protocols may influence both treatment approaches and outcomes, thereby limiting the extent to which our results reflect broader clinical practice and are applicable to other settings.

Although this study focused on the acute admission period, it is well recognised that paediatric hand burns carry significant risk for both short- and long-term functional, aesthetic, and psychological sequelae [19,21,22,23,24,25,26,27,28,43]. Our findings do not capture these longer-term outcomes, nor do they reflect the broader population of children managed in outpatient settings. Future research—ideally through longitudinal cohort studies—should address these gaps by including outpatient cases and stratifying long-term outcomes by hand involvement. Doing so would enhance the generalisability of findings and provide a more complete understanding of recovery trajectories. Such research could also determine whether hand involvement serves as an independent prognostic factor in the long-term, ultimately guiding more evidence-based assessment, referral, and management strategies in paediatric burn care.

## 5. Conclusions

Hand burns are common in children and may result in significant and lasting impairments in function and appearance. Although the epidemiology, burn characteristics, and disposition of paediatric hand burns are well-defined in the literature, our study was the first to investigate differences in these parameters according to hand involvement. Children with burns isolated to the hands were younger, had smaller %TBSA estimates, were more prone to contact and friction burns, and were more likely to undergo isolated skin grafting compared to children with burns involving the hands and other sites and children with burns not involving the hands. Despite these distinctions, multivariate regression analysis demonstrated that hand involvement was not an independent predictor of length of stay in paediatric burn patients.

Future research incorporating outpatient data and investigating long-term surgical and rehabilitation requirements based on hand involvement would provide a more comprehensive understanding of its prognostic significance. Such studies could further inform evidence-based management strategies and improve outcomes for paediatric patients with burn injuries.

## Figures and Tables

**Table 1:** Descriptive Statistics and Group Comparisons for Children Admitted to the Paediatric Burns Service of Western Australia for >24 h or for Burn Wound Management in Theatre (1 July 2012 to 30 June 2022).

Variable		All Patients (n = 2196)	Hand Only (n = 241)	Hand and Other Sites (n = 274)	Other Sites Only (n = 1681)	$X^{2}(df)$	p
Sex, n (%)	*Male*	1314 (60%)	150 (62%)	156 (57%)	1008 (60%)	1.55(2)	0.460
	*Female*	882 (40%)	91 (38%)	118 (43%)	673 (40%)		
Age at Injury, median (IQR) range	*Years*	3.2 (1.5–8.6) 0–17	2.3 (1.1–4.3) 0–17	3.6 (1.5–9.4) 0.1–16	3.4 (1.5–8.9) 0–17	27.7(2)	<0.001
Area of Residence *, n (%)	*Metropolitan*	1448 (66%)	169 (70%)	162 (59%)	1117 (66%)	7.92(4)	0.095
	*Country*	729 (33%)	71 (29%)	108 (39%)	550 (33%)		
	*Overseas*	15 (0.7%)	1 (0.4%)	3 (1%)	11 (0.7%)		
	*Other* */Unknown	4 (0.2%)	0 (0%)	1 (0.4%)	3 (0.2%)		
Mechanism of Injury ^†^, n (%)	*Scald*	1106 (50%)	50 (21%)	100 (37%)	956 (57%)	295(8)	<0.001
	*Contact*	548 (25%)	95 (39%)	83 (30%)	370 (22%)		
	*Flame*	191 (9%)	10 (4%)	58 (21%)	123 (7%)		
	*Friction*	197 (9%)	72 (30%)	23 (8%)	102 (6%)		
	*Other* ^†^	139 (6%)	13 (5%)	7 (3%)	119 (7%)		
	*Not Documented*	15 (0.7%)	1 (0.4%)	3 (1%)	11 (0.7%)		
Burn Depth, n (%)	*Superficial Dermal*	130 (6%)	7 (3%)	18 (7%)	105 (6%)	10.6(6)	0.100
	*Mid-Dermal*	621 (28%)	61 (25%)	72 (26%)	488 (29%)		
	*Deep Dermal*	506 (23%)	64 (27%)	65 (24%)	377 (22%)		
	*Full Thickness*	251 (11%)	37 (15%)	30 (11%)	184 (11%)		
	*Not Documented*	688 (31%)	72 (30%)	89 (32%)	527 (31%)		
TBSA, median (IQR) range	*Percentage body surface area*	2 (1–4) 0.1–90	1 (0.5–1) 0.1–3	2.5 (1.5–4) 0.1–90	2 (1–4) 0.1–46	267(2)	<0.001
Burn Management Procedure in Theatre ^‡^, n (%)	*Yes*	1212 (55%)	148 (61%)	156 (57%)	908 (54%)	5.05(2)	0.080
	*No*	984 (45%)	93 (39%)	118 (43%)	773 (46%)		
Procedure Type, n (%)	*ReCell*^®^ *Only*	560 (26%)	51 (21%)	75 (27%)	434 (26%)	47.6(4)	<0.001
	*Skin Graft Only*	184 (8%)	48 (20%)	10 (4%)	126 (8%)		
	*ReCell* ^®^ * + Skin Graft*	437 (20%)	44 (18%)	69 (25%)	324 (19%)		
	*Other Procedure/* *Not Documented* ^‡^	31 (1%)	5 (2%)	2 (0.7%)	24 (1%)		
	*No Procedure*	984 (45%)	93 (39%)	118 (43%)	773 (46%)		
Intensive Care Unit Admission, n (%)	*Yes*	20 (0.9%)	0 (0%)	6 (2%)	14 (0.8%)	7.3(2)	0.026
	*No*	2176 (99%)	241 (100%)	268 (98%)	1667 (99%)		
Length of Stay, median (IQR) range	*No*	3 (1.5–6.4) 0.1–77	1.8 (0.5–3.8) 0.2–31	3.6 (1.7–7.1) 0.1–68	3.3 (1.6–6.6) 0.21–77	62.3(2)	<0.001

* Other geographical locations include overseas and no fixed abode. ^†^ Other mechanisms of burn injury include chemical, electrical, radiant heat, cooling, pressurised gas/air, and other specified causes. ^‡^ Other burn management procedures in theatre include escharotomy, debridement only, use of temporary skin closure product, use of dermal reconstructive product, allograft, dressing change, fasciotomy, amputation, and other specified causes.

**Table 2:** Univariate Regression Analysis of the Association Between Study Group and Length of Hospital Stay.

Variable		Coef	Lower 95% CI ^†^	Upper 95% CI ^†^	p
Group	Hand + other sites	0.78	0.55	1.02	<0.001 ***
	No hand burn	0.57	0.40	0.74	<0.001 ***

Note. The reference category for this analysis was Group 1 (hand-only burns). ^†^ Confidence interval. *** *p* < 0.001.

**Table 3:** Final Multivariable Model Assessing the Association Between Patient and Injury Factors and Length of Hospital Stay.

Variable		Coef	Lower 95% CI	Upper 95% CI	*p* > |z|
Group	Hand + other sites	−0.08	−0.30	0.13	0.458
	No hand burn	−0.05	−0.24	0.14	0.577
Mechanism of Injury	Flame	0.36	0.23	0.49	<0.001 ***
Area of Residence	Country	0.41	0.32	0.50	<0.001 ***
TBSA	TBSA (log)	0.45	0.39	0.51	<0.001 ***
Burn Management Procedure	ReCell^®^ and graft	0.33	0.23	0.43	<0.001 ***

Note. The reference categories for this analysis were as follows: Group = Group 1 (hand-only burns); burn type = scald; area of residence = metropolitan; burn management procedure = no surgery. *** *p* < 0.001.

## Data Availability

The data are not publicly available due to HREC privacy requirements. The de-identified data presented in this study are available on request from the corresponding author.

## Supplementary Materials

The following supporting information can be downloaded at: https://www.mdpi.com/article/10.3390/ebj7020023/s1, Table S1: Univariate regression analysis of the association between study variables and length of hospital stay; Table S2: Backward elimination and multivariate regression analysis of the association between study variables and length of hospital stay.

## Abbreviations

The following abbreviations are used in this manuscript:

LOS	Length of stay
BSWA	Burn Service of Western Australia
TBSA	Total body surface area
ICU	Intensive care unit
PCH	Perth Children’s Hospital
PMH	Princess Margaret Hospital
CAHS	Child and Adolescent Health Service
STROBE	Strengthening the Reporting of Observational Studies in Epidemiology
IQR	Interquartile range
M	Mean
SD	Standard deviation
CI	Confidence interval
AIC	Akaike Information Criterion
BIC	Bayesian Information Criterion
HREC	Human Research Ethics Committee
