# Supplementary material for: Hand Involvement and Its Association with Burn Characteristics, Surgical Management, and Length of Stay in Paediatric Inpatients: A 10-Year Cross-Sectional Study from Western Australia
Source: Eur Burn J. 2026 Apr 30;7(2):23. doi: 10.3390/ebj7020023 (PMC13214700; doi:10.3390/ebj7020023)
Supplement: Supplementary file 1 [file ebj-07-00023-s001.zip › ebj-4193587-supplementary.pdf]

Supplementary Material

Table S1: Univariate regression analysis of the association between study variables and length of hospital stay

For the univariate regression analyses, the following Stata coding was used:

```
glm los x, family(nbinomial) link(log) robust nolog
where x = test variable
```

This generated the following information:

Table S1: Univariate Regression Analysis of the Association Between Study Variables and Length of Hospital Stay

Variable		Coef	Lower 95% CI*	Upper 95% CI*	P >|z|
**Group**	*Hand + other sites*	0.78	0.55	1.02	< .001
	No hand burn	0.57	0.40	0.74	< .001
**Sex**	*Male*	0.02	-0.10	0.13	0.76
**Age**		0.57	-0.01	0.02	0.48
**Area of Residence**	*Country*	0.38	0.27	0.50	< .001
	Overseas	-0.18	-0.53	0.16	0.30
**Mechanism of Injury**	*Contact*	-0.19	-0.31	-0.07	0.002
	Flame	0.73	0.54	0.93	< .001
	Friction	-0.34	-0.53	-0.14	0.001
	Other	-0.31	-0.48	-0.14	< .001
**Burn Depth**	*Mid-Dermal*	-0.15	-0.31	0.02	0.08
	Deep Dermal	-0.01	-0.19	0.28	0.95
	Full Thickness	0.45	0.21	0.70	< .001
**TBSA (log)**		0.46	0.41	0.52	< .001
**Burn Management Procedure**	*Skin Graft Only*	-0.34	-0.57	-0.10	0.006
	ReCell® + Skin Graft	0.55	0.39	0.71	< .001
	No Procedure	-0.23	-0.33	-0.13	< .001
	Other/Not Documented	0.08	-0.26	0.41	0.65

Note. The reference categories for this analysis were as follows: Group = Group 1 (hand-only burns); area of residence = metropolitan; mechanism of injury = scald; burn depth = superficial dermal; burn management procedure = ReCell® only.
*Confidence interval

Table S2: Backward elimination and multivariate regression analysis of the association between study variables and length of hospital stay

For the backward elimination and multivariate regression analyses, the following Stata coding was used:

```
stepwise, pr(0.05) lockterm1: glm los (i.group) log_tbsa i.cause2 i.surg_type i.res_area, family(nbinomial) link(log) robust nolog
```

```
1b.cause2 dropped because of estimability
1b.surg_type dropped because of estimability
1b.res_area dropped because of estimability
begin with full model
p = 0.8263 >= 0.0500  removing 4.cause2
p = 0.6396 >= 0.0500  removing 4.surg_type
p = 0.4190 >= 0.0500  removing 5.cause2
p = 0.3940 >= 0.0500  removing 2.surg_type
p = 0.2546 >= 0.0500  removing 5.surg_type
p = 0.2466 >= 0.0500  removing 3.res_area
p = 0.1408 >= 0.0500  removing 2.cause2
```

This generated the following information:

Table S2. Final Multivariable Model Assessing the Association Between Patient and Injury Factors and Length of Hospital Stay

**Variable**		Coef	Lower 95% CI	Upper 95% CI	P >|z|
**Group**	*Hand + other sites*	-0.08	-0.30	0.13	0.458
	No hand burn	-0.05	-0.24	0.14	0.577
**Mechanism of Injury**	*Flame*	0.36	0.23	0.49	< .001***
**Area of Residence**	*Country*	0.41	0.32	0.50	< .001***
**TBSA**	*TBSA (log)*	0.45	0.39	0.51	< .001***
**Burn Management Procedure**	*ReCell® and graft*	0.33	0.23	0.43	< .001***

Note. The reference categories for this analysis were as follows: Group = Group 1 (hand-only burns); burn type = scald; area of residence = metropolitan; burn management procedure = no surgery.
*p < 0.05; **p < 0.01; ***p < 0.001
